# Impact of Propofol Bolus Administration on the Nociceptive Flexion Reflex Threshold and Bispectral Index in Children—A Case Series

**DOI:** 10.3390/children8080639

**Published:** 2021-07-27

**Authors:** Maximilian David Mauritz, Felix Uhlenberg, Eik Vettorazzi, Chinedu Ulrich Ebenebe, Dominique Singer, Philipp Deindl

**Affiliations:** 1 Department of Neonatology and Pediatric Intensive Care Medicine, University Children’s Hospital, University Medical Center Hamburg-Eppendorf, 20251 Hamburg, Germany; f.uhlenberg@kh-itzehoe.de (F.U.); c.ebenebe@uke.de (C.U.E.); d.singer@uke.de (D.S.); p.deindl@uke.de (P.D.); 2 Department of General Pediatrics and Adolescent Medicine, Children’s and Adolescents’ Hospital Datteln, Witten/Herdecke University, 45711 Datteln, Germany; 3 Department of Pediatrics and Adolescent Medicine, Neonatology and Pediatric Intensive Care Medicine, Itzehoe Medical Center, 25524 Itzehoe, Germany; 4 Department of Medical Biometry and Epidemiology, University Medical Center Hamburg-Eppendorf, 20251 Hamburg, Germany; e.vettorazzi@uke.de

**Keywords:** propofol, bispectral index, pain, sedation, nociceptive flexion reflex threshold

## Abstract

We analyzed the impact of propofol administration during continuous sedation and analgesia on the nociceptive flexion reflex threshold (NFRT) and Bispectral Index (BIS) in ventilated children. We examined patients who received propofol before planned endotracheal suctioning. Patients were clinically assessed using the modified Face, Legs, Activity, Cry, Consolability (mFLACC) scale and COMFORT-B (Comfort Behavior) scale. We continuously recorded the NFRT and BIS. We recorded 23 propofol administrations in eight patients with an average age of 8.6 ± 3.5 years. The median (minimum-maximum) scores for the mFLACC scale and COMFORT-B scale were 0 (0–5) and 6 (6–17), respectively, before the bolus. The administration of a weight-adjusted propofol bolus of 1.03 ± 0.31 mg/kg resulted in an increase in NFRT and burst-suppression ratio; BIS and electromyogram values decreased. Changes from baseline (95% CI) after propofol bolus administration were BIS −23.9 (−30.8 to −17.1), EMG -10.5 dB (−13.3 to −7.7), SR 14.8 % (5.6 to 24.0) and NFRT 13.6 mA (5.5 to 21.7). Further studies are needed to determine whether sedated children may benefit from objective pain and sedation monitoring with BIS and NFRT.

## 1. Introduction

Critically ill children experience numerous stressful and potentially painful procedures during intensive care treatment [1]. In addition to benzodiazepines, propofol is widely used as a sedative agent in pediatric intensive care units (PICU). In addition to continuous infusion for short-term sedation, it is used as a bolus administration for short-term interventions, care facilitation, or extubation [2,3]. Propofol is also frequently used in German intensive care units to facilitate stressful procedures in children, such as endotracheal suctioning [4]. There are no available data in the literature on the use of boluses of propofol in these indications for other countries. It is unclear how additional propofol bolus administrations during continuous sedation and analgesia impact pain reflex thresholds and electroencephalography (EEG) based sedation parameters in children.

### 1.1. Clinical Assessment of Pain and Sedation

It is ideal to avoid painful or stressful procedures in children in the PICU. Nevertheless, if such procedures are unavoidable, then adequate pain management and sedation are paramount. The assessment of pain and sedation in the PICU is a continuing challenge. International guidelines for the intensive care treatment of children [5,6,7] recommend adequate analgesia and sedation based on self-reports [8] and the use of clinical scales when self-reports are not feasible due to preverbal age, cognitive impairment, or impaired communication, for example, due to endotracheal intubation [5].

For clinical assessment of sedation and pain, the Face, Legs, Activity, Cry, Consolability (FLACC) and the COMFORT-B scale are most widely used on PICUs today, respectively [9]. A FLACC score of >2 indicates that the patient is experiencing pain [10]. The “modified FLACC” (mFLACC) is adapted for intubated pediatric patients [11]. Adequate sedation according to COMFORT-B ranges from 11 to 22; values below and above indicate under- and oversedation, respectively [12].

### 1.2. Objective Monitoring of Pain and Sedation

Currently, no technology for measuring pain and sedation in children in the PICU has been widely accepted for clinical use [13]. The polysynaptic spinal nociceptive flexion reflex threshold (NFRT) is a technique that allows the estimation of the degree of analgesia [14]. Nonetheless, this novel analgesia monitor has not yet been validated in pediatric patients.

By applying electrical stimuli to the sural nerve through surface electrodes, the nociceptive flexion reflex (NFR) of the ipsilateral biceps femoris muscle can be measured utilizing a surface electromyogram (EMG). The EMG amplitude of the NFR correlates with the intensity of subjective pain sensation in adults [15]. The stimulus intensity associated with a 50% probability to elicit a reflex response is defined as the NFRT. In awake patients, the applied stimuli are perceived from not painful to slightly above the pain threshold. The NFRT correlates with the subjective pain threshold of adults [16] and thus can be used as an objective value for the threshold of nociception [14]. NFRT is influenced in a dose-dependent manner by the concentration of opioids, inhalational anesthetics, and propofol in adult patients [17,18,19]. Regarding the NFRT, there are no published target values or limits for adequate analgesia in children.

The Bispectral Index (BIS) is the most widely used EEG-based sedation and hypnosis monitor in adults. Developed originally for adults, the BIS was investigated in several studies to estimate sedation levels in infants and children with promising results [20,21]. The BIS is computed by an unpublished algorithm as a number between 0 (no brain activity) and 100 (awake), using a combination of bispectral EEG analysis, the EEG burst-suppression ratio (SR), and facial EMG data. Lamas and Lopez-Herce recommend BIS values in the range of 60–80 for stable children and 40–60 for unstable children on mechanical ventilation [22]. In a comprehensive study, Malviya et al. evaluated the BIS in children by clinical sedation level and found a mean BIS value of 65 for their lowest sedation level category [23]. BIS values below the above suggest over-sedation of the patient, according to the authors. It should be noted that the BIS is not a validated procedure for sedation monitoring in children. In adults, there is insufficient evidence on the effects of BIS monitoring compared to clinical assessment of sedation in critically ill patients on mechanical ventilation regarding clinical outcomes [24].

International guidelines promote an individual sedation goal for each patient and regular re-evaluation of this goal [5,6,7]. Influencing factors include the patient’s medical condition, circulatory and ventilatory situation, age, and environmental factors. Sedative and analgesic therapy should be titrated according to effect. With suitable clinical assessment scales, a regular re-assessment of the current sedation should be performed.

We recently published results from the “PredIction of Nociception in CHildren” PINCH study, in which we combined continuous BIS and NFRT monitoring with clinical scales to predict noxious responses to endotracheal suctioning in ventilated children [25]. Patients were excluded from the PINCH study prediction analysis if they had received a propofol bolus within 10 min prior to endotracheal suctioning. This report presents a secondary analysis of those patients excluded from the PINCH study.

We aimed to describe the impact of additional propofol bolus administration during continuous sedation and analgesia on the NFRT and BIS in ventilated children.

## 2. Materials and Methods

### 2.1. Patients

After obtaining approval from the local ethics committee (Approval PV5210, Ethikkommission der Ärztekammer Hamburg, Germany, 30 March 2016) and written informed consent from both parents, a prospective observational study was performed of mechanically ventilated children in the 14-bed PICU of the University Children’s Hospital, University Medical Center Hamburg Eppendorf, Germany between 10 March 2017, and 24 September 2018. According to the local standard protocol, patients received continuous infusions of a combination of midazolam for sedation and an opiate for analgesia. For short post-procedural or short-term post-operative sedation, propofol was infused alone or in combination with an opioid. When clinically indicated, patients also received esketamine or clonidine. Patients excluded from the PINCH study because of bolus administration of propofol are reported in this case series [25]. Exclusion criteria included neuromuscular block, neuromuscular diseases, or trauma to the peripheral or central nervous system. According to the nurse’s clinical judgment, of the 30 initially recruited patients, eight received a weight-adjusted bolus of propofol before endotracheal suctioning.

### 2.2. Study Protocol

The nurse in charge and an independent observer (M.D.M., F.U.) clinically assessed the patient using the modified Face, Legs, Activity, Cry, Consolability (mFLACC) scale [11,26] and the COMFORT-B scale [27,28] after a rest period without intervention or disturbance within the preceding 30 min. PICU staff had been trained in the use of the mFLACC scale prior to the study. The COMFORT-B scale was already implemented as part of the local treatment standard. In addition, we continuously recorded the NFRT and BIS. The NFRT was determined using a Paintracker instrument (Dolosys GmbH, Berlin, Germany). The Paintracker uses an automated threshold tracking system that repeatedly applies an electrical stimulation at intervals of 10 s to the sural nerve and records the electromyographical reflex response of the ipsilateral biceps femoris muscle [29]. The Paintracker automatically alters the electrical stimulation current to determine the NFRT [16,30]. We recorded the BIS on a 1-s time scale using the BIS VISTA Monitoring System (Software Revision 1.15, Medtronic, Dublin, Ireland) with pediatric electrodes (BIS Pediatric Sensor, Medtronic, Dublin, Ireland). The BIS was also used to determine the SR and EMG, each of which was provided by the BIS monitor as the percentage of isoelectric EEG over the last 63 s and as absolute power in the range 70–110 Hz, expressed in decibels (dB), relative to 0.0001 μV2. The nursing staff and independent observers were both blinded to the BIS and NFRT monitors. We applied averaging to determine NFRT and BIS values at the baseline time t0 (−30 to 0 s) and time t1 (120 to 180 s) relative to propofol injection.

### 2.3. Statistical Analysis

We analyzed the impact of propofol bolus administration on the NFRT, BIS, EMG activity, and SR. We report continuous variables as the mean ± standard deviation (SD) or median with interquartile range (IQR) and categorical variables as category counts and percentages. Boxplots are displayed as quartiles with 95% confidence intervals (CIs). Changes in BIS; NFRT, SR and EMG were assessed using a paired-samples T-test and paired Wilcoxon signed-rank test. *p*-values less than 0.05 were considered significant. Statistical analyses were performed using Python (Python Software Foundation, Beaverton, OR, USA) and R 3.6.3 (R Core Team, Vienna, Austria).

## 3. Results

### 3.1. Patient Characteristics

We analyzed 23 cases of propofol bolus administration with a median (minimum–maximum) administration number of 3 (1–6) per patient in eight patients. Patients were admitted for various reasons to the PICU. Patients were 2.5, 4.9, 6.8, 9.4, 10.3, 10.9, 11.9, and 12.3 years old (mean 8.6 ± 3.5 years). They were on mechanical ventilation for a total of 7.9 ± 5.5 days (Table 1). None of the patients died during their course of treatment in the PICU.

### 3.2. Impact of Propofol on BIS and NFRT

**Figure 1:**
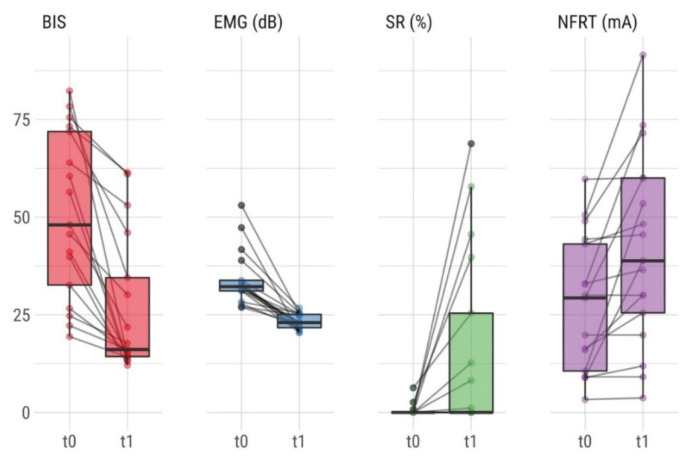
Boxplot showing the distribution of bispectral index (BIS), electromyogram (EMG), suppression ratio (SR), and nociceptive flexion reflex threshold (NFRT) before (t0) and after (t1) propofol bolus administration. Horizontal lines indicate changes during individual measurements.

**Figure 2:**
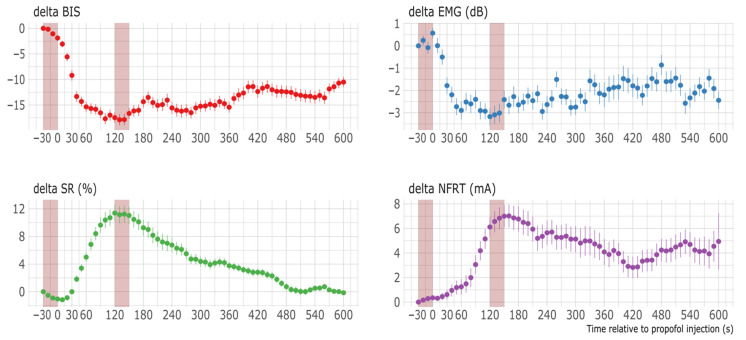
Changes from baseline values (delta) of Bispectral Index (BIS), electromyogram (EMG), suppression ratio (SR), and nociceptive flexion reflex threshold (NFRT) over time with respective 95% CIs. Propofol was administered at 0 s. The red areas mark the periods t0 and t1, in which the values for BIS, EMG, SR, and NFRT were averaged.

The mean ± SD administered propofol dose was 1.03 ± 0.31 mg/kg (range 0.48–2.04 mg/kg) in these observations. The median (minimum–maximum) clinical pain and sedation score assessed by the independent observer before the propofol bolus (t0) was 0 (0–5) for the mFLACC scale and 6 (6–17) for the COMFORT-B scale. The baseline values (median, IQR) of the sedation and pain monitoring were BIS 51.9, IQR 31.1–72.3; EMG activity 32.3 dB, IQR 30.4–39.5 dB; SR 0%, IQR 0–0% and NFRT 29.7 mA, IQR 10.2–45.5 mA, respectively. After propofol bolus administration (t1), the BIS, EMG, SR, and NFRT values (median, IQR) were 16.9, IQR 14.3–41.6; 23.1 dB, IQR 21.5–25.3 dB; 0%, IQR 0–29 % and 42.2 mA, IQR 24.1–62.9 mA, respectively (Figure 1). Both BIS and EMG values decreased within 1 min following propofol bolus administration, whereas the SR and NFRT peaked 2 min after the propofol bolus. Changes from baseline (95% CI) after propofol bolus administration (t1) were −23.9 (−30.8 to −17.1), −10.5 (−13.3 to −7.7), 14.8 (5.6 to 24.0) and 13.6 (5.5 to 21.7) for BIS, EMG, SR, and NFRT respectively. There was a statistically significant difference (*p* < 0.05) between all values before (t0) and after propofol administration (t1). Changes from baseline values over time are shown in Figure 2 with respective 95% CIs. We assessed the changes in parameters across the different time points using a paired Wilcoxon signed-rank test. The analysis showed a strong effect for BIS (z = −4.2, *p* < 0.01, n = 23, effect size r = −0.87), EMG (z = −4.2, *p* < 0.01, n = 23, r = −0.98), SR (z = −3.1, *p* < 0.01, n = 23, r = −0.72) and NFRT (z = −3.3, *p* < 0.01, n = 23, r = −0.77).

## 4. Discussion

Propofol bolus administration had a significant impact on both the NFRT and BIS in ventilated children. After propofol injection, the NFRT and SR increased, and the BIS and EMG decreased.

Based on their clinical judgment, each nurse in charge determined whether a bolus dose of propofol should be administered, as intended in the original study protocol. The combination of low BIS values (median 51.9, IQR 31.1–72.2) and low COMFORT-B scores (median; minimum–maximum: 6; 6–17) indicates that patients were already profoundly sedated before they received the additional propofol bolus. Consequently, we observed very low BIS values (median 16.9, IQR 14.3–41.6) and a high amount of burst suppressions after propofol administration, suggesting very deep sedation of the patients [22,23].

The assessment of adequate sedation and analgesia, especially in pediatric patients, is challenging. Clinical judgment is strongly dependent on the assessor’s experience, and even when clinical scales are used, there is a relevant risk of bias [31]. In addition, pain experiences correlate with the development of chronic pain syndromes, post-traumatic stress disorder (PTSD), and low health-related quality of life [7]. Although over-sedation poses a considerably greater risk here, under-sedation has also long been suspected of promoting PTSD. In this context, caregivers tend to avoid under-sedation, especially in children. This intention, in turn, may increase the risk of over-sedation, especially in already deeply sedated patients [32].

We observed a significant effect of propofol on the NFRT in ventilated children. Opioids increase the NFRT in a dose-dependent manner, reflecting the spinal analgesia level. Propofol has a similar effect on the NFRT in adults [33]. This effect has not yet been described in children. Propofol is often used for short-term sedation in PICUs [2,3,7] and procedural sedation inside and outside the intensive care unit [34,35]. Despite its lack of analgesic effect, it is also frequently used in German PICUs to facilitate stressful procedures, such as endotracheal suctioning [4]. In adults, it has been shown to possibly suppress neurons in the ventral spinal cord, resulting in increased NFRT [36]. Ongoing research investigates whether propofol influences peripheral (dorsal horn) and central nociception or whether it only suppresses the motor reflex response to a painful stimulus [19]. The absence of movement response to a painful intervention cannot always be interpreted as a lack of nociception [14]. On the contrary, also under high doses of propofol, including burst suppression EEG and high NFRTs, cortical responses have been provoked during intense noxious stimuli [37].

We also observed a significant decrease in BIS values after propofol administration in our patients. With increasing anesthetic dosage, the high-frequency EEG components decrease, and the low-frequency components increase, indicating central nervous system attenuation. This effect is primarily reflected in the BetaRatio and SynchFastSlow analysis of the BIS algorithm [38], explaining the decrease in BIS values in our patients. Since the original algorithm is unpublished, it is unclear whether the BIS algorithm also uses EMG activity as a surrogate parameter in certain areas. Other studies have demonstrated similar effects on the BIS [39]. The presence of burst suppression during sedation has been shown to correlate with delirium risk, prolonged hospital stay, cognitive impairment after treatment, and increased mortality in adults [40]. Recent studies investigating risk factors for emergence delirium in pediatric patients could not confirm a relationship between intra-operative burst suppression and post-operative delirium [41]. However, studies regarding the effects of deep sedation in intensive care patients are lacking.

Each nurse was blinded to the BIS and NFRT measurements in our study and decided whether an additional propofol bolus should be administered before endotracheal suctioning based on their clinical judgment alone. This approach resulted in very deep sedation in our patients. Concerns that a potentially unpleasant maneuver such as endotracheal suctioning might result in the child’s arousal might have overruled the nurses’ clinical patient assessment.

EEG-based sedation monitoring may help to distinguish deep sedation from over-sedation by reflecting brain activity. The same is true for the analgesia level reflected by the NFRT. We observed that in children with low mFLACC pain scores, including an absent pain response to stimulation, the NFRT still increased after propofol bolus administration. Hence, further studies in objective monitoring of sedation and pain are needed to validate their use in capturing analgesia and sedation regions that are not accessible by clinical scores. Nevertheless, these tools may offer a potential advantage for managing pain and sedation in children treated in the PICU.

However, especially in deeply sedated children, who cannot express themselves, assessments based on clinical scales alone can be imprecise [21]. Therefore, randomized controlled trials should explore whether objective and accurate pain and sedation monitoring can potentially improve PICU patient outcomes, especially in avoiding over-sedation and its potential adverse effects.

### Limitations

The sample of patients recruited in this study was small and heterogeneous regarding age, underlying disease, and medication. Half of the patients shown in this case series were admitted to the PICU postoperatively; this sample does not represent the broad spectrum of a pediatric intensive care unit. We performed multiple measurements in the patients, which were considered in the statistical analysis using paired-samples T-test and paired Wilcoxon signed-rank test. We defined the time point after propofol injection (t1) as the period of 120–150 s after administration. This definition was based on the clinical pharmacokinetics of propofol and corresponds with maximal BIS change after propofol injection in children for this period [39]. Due to the original study’s design, we did not interview the nursing staff about the factors that motivated their decision to administer propofol as a bolus. Further studies are needed to determine whether sedated children may benefit from objective and accurate pain and sedation monitoring.

## 5. Conclusions

Based on the clinical judgment of nurses, ventilated children in our sample received a propofol bolus prior to endotracheal suctioning. Propofol injection significantly increased the NFRT and decreased the BIS, with a high SR and low EMG activity. Only the baseline values of BIS and NFRT had a significant impact on the respective values after propofol injection. Both tools are not yet validated for monitoring pain and sedation in the PICU, and further studies are needed to validate their use.

## Figures and Tables

**Table 1:** Clinical characteristics of the included patients.

Characteristics	*n* = 8
Male	5 (62.5)
Female	3 (37.5)
Age, years	8.6 ± 3.5
Bodyweight, kg	24.0 ± 8.9
Time on mechanical ventilation, days	7.9 ± 5.5
Length of PICU stay, days	14.1 ± 9.7
Mode of mechanical ventilation	
SIMV	6 (75)
BIPAP	2 (25)
Diagnostic group	
Non-cardiac post-operative	4 (50)
Miscellaneous (including injury)	3 (37.5)
Cardiac post-operative	1 (12.5)

PICU: pediatric intensive care unit; SIMV: synchronized intermittent mandatory ventilation; BIPAP: bilevel positive airway pressure. Continuous variables are shown as the mean ± standard deviation, counts as *n* (% of all included patients).

## Data Availability

The data presented in this study are available on request from the corresponding author. The data are not publicly available due to privacy restrictions.
